# An Activating Mutation Reveals a Second Binding Mode of the Integrin α2 I Domain to the GFOGER Motif in Collagens

**DOI:** 10.1371/journal.pone.0069833

**Published:** 2013-07-29

**Authors:** Federico Carafoli, Samir W. Hamaia, Dominique Bihan, Erhard Hohenester, Richard W. Farndale

**Affiliations:** 1 Department of Life Sciences, Imperial College London, London, United Kingdom; 2 Department of Biochemistry, University of Cambridge, Downing Site, Cambridge, United Kingdom; Griffith University, Australia

## Abstract

The GFOGER motif in collagens (O denotes hydroxyproline) represents a high-affinity binding site for all collagen-binding integrins. Other GxOGER motifs require integrin activation for maximal binding. The E318W mutant of the integrin α2β1 I domain displays a relaxed collagen specificity, typical of an active state. E318W binds more strongly than the wild-type α2 I domain to GMOGER, and forms a 2:1 complex with a homotrimeric, collagen-like, GFOGER peptide. Crystal structure analysis of this complex reveals two E318W I domains, A and B, bound to a single triple helix. The E318W I domains are virtually identical to the collagen-bound wild-type I domain, suggesting that the E318W mutation activates the I domain by destabilising the unligated conformation. E318W I domain A interacts with two collagen chains similarly to wild-type I domain (high-affinity mode). E318W I domain B makes favourable interactions with only one collagen chain (low-affinity mode). This observation suggests that single GxOGER motifs in the heterotrimeric collagens V and IX may support binding of activated integrins.

## Introduction

The integrins are heterodimeric adhesion receptors, with 24 permitted pairings selected from 18 α and 8 β subunits [1]. Integrin α2β1 is the best-researched of the four collagen-binding integrins, which share with the closely-related leukocyte β2 integrins the presence of an autonomously-folding VWF A, or inserted (I), domain that contains the primary ligand-binding site [2]. The I domain, which adopts the Rossman-fold [3], is located between blades 2 and 3 of the α subunit β-propeller and believed to interact at its base with the β subunit I-like domain, a similar structure that serves as the primary ligand-binding site in integrins without an α subunit I domain. In all integrins, a series of loops at the distal surface of these I domains bind a Mg^2+^ ion that is crucially involved in the binding of ligand [4]. This arrangement is known as the metal ion-dependent adhesion site (MIDAS).

The integrins are bi-directional signalling receptors [5]: ligand binding to the extracellular region of integrins signals to the interior of the cell, and integrin affinity for ligand can be increased by stimulation of the cell through other pathways. Upon activation, the gross conformation of the extracellular region of integrins changes from a bent to an upright posture (reviewed in [5], [6]), allowing unimpeded access to the ligand-binding head of the receptor. Since integrin ligands are generally macromolecular structures, this is an important aspect of the activation process, but its basis remains incompletely understood.

The I domain conformation is also mobile [7]. The I domain consists of a parallel β-sheet surrounded by α-helices. The elucidation of ligated and free forms of the α2 I domain revealed that, upon ligation, helix 7 travels downwards by ∼10 Å, away from the MIDAS [8], [9]. This provides a bidirectional conduit for transmission of information. In the high-affinity form of the integrin, a glutamic acid in the linker segment following helix 7 is believed to interact with the β subunit MIDAS [10], [11]. A second mobile feature of the I domain is the C-helix, a short α-helix close to the MIDAS that prevents ligation of the resting integrin. The C-helix is stabilised by a salt bridge between an arginine at its N-terminus and a glutamic acid at the top of helix 7 (R288 and E318 in the α2 I domain). Upon ligation (or activation) the salt bridge is broken by downwards motion of helix 7, and the C-helix is remodelled to an additional turn in helix 6 [9].

Conformational change in the α2 I domain was revealed by its co-crystallisation with a collagen-like, triple-helical, peptide of sequence [GPO]_2_GFOGER[GPO]_3_, O denoting hydroxyproline [9]. In this co-crystal, the glutamate carboxylate anion of the GER triplet co-ordinates the bound Mg^2+^ ion directly, leading to a rearrangement of the ion’s octahedral co-ordination shell. Additional contacts with the α2 I domain are made by phenylalanine and arginine residues of the GFOGER peptide. GFOGER constitutes a high-affinity motif for all the collagen-binding integrins reported to date [12]–[14]. However, cellular activation is reported to be necessary for full binding to related, lower-affinity motifs. The interaction of platelets through α2β1 with short triple-helical peptides containing GMOGER, for example, required activation with ADP before full platelet binding was achieved [15]. Similarly, the activatory monoclonal antibody TS2/16 increased platelet binding to longer triple-helical Toolkit peptides containing GLOGER, GMOGER and other sub-optimal motifs [16]. Platelet adhesion to a GFOGER-containing peptide was not significantly affected in either study, showing that the phenylalanine sidechains of triple-helical GFOGER confer high-affinity binding.

Various strategies have been adopted to produce constitutively active or inactive forms of the α2 I domain. Inactivation has been achieved by mutating the Mg^2+^ ligand T221 to alanine, disrupting the MIDAS [16], [17], and by introducing a disulphide bridge between helices 1 and 7 such that helix 7 is locked into the low-affinity (“closed”) conformation (Hamaia et al., in preparation). Activation has been achieved by disulphide-locking helix 7 into the high-affinity (“open”) conformation that is seen in the GFOGER-I domain co-crystal structure [15]. An alternative strategy was used by Tuckwell, who replaced E318 with tryptophan, resulting in a destabilised C-helix and a MIDAS that was more open to ligation [18]. A recent crystal structure of the analogous E317A mutant of the α1 I domain was reported by the group of Heino [19]. In their unligated structure, the C-helix was unwound, but helix 7 remained in the low-affinity conformation.

Here we report a functional and structural characterisation of the α2 I domain E318W mutant. Unlike the wild-type I domain, the E318W mutant binds the GFOGER peptide with 2:1 stoichiometry. Because the three chains of a collagen triple helix are not topologically equivalent, two distinct binding modes are observed crystallographically, one resembling that of the wild-type I domain and the other sacrificing one of the two favourable contacts with the phenylalanine residues of the GFOGER motifs. The structure suggests how activated integrin α2β1 might bind to heterotrimeric collagens harbouring a high-affinity motif in only one of the three chains of the triple helix.

## Results

### Binding of Integrin α2 I E318W to Immobilised Triple-helical Peptides

**Figure 1:**
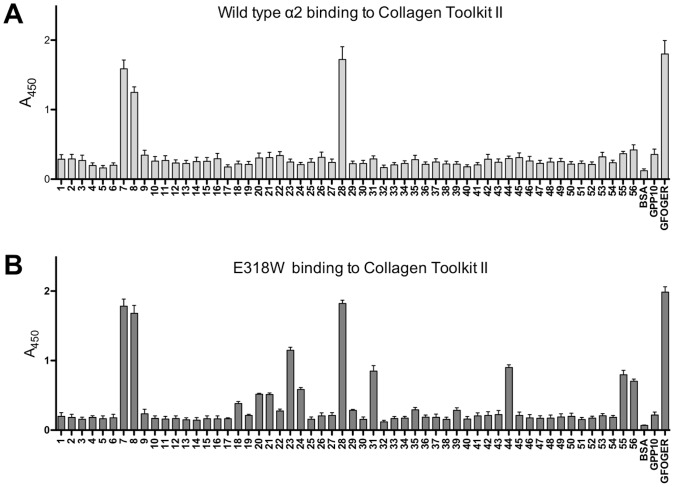
Toolkit II analysis of the integrin α2 I domain E318W mutant. I domain binding, detected using HRP-linked anti-GST, to immobilised Toolkit II peptides was performed as described in Materials and Methods. BSA, and the triple-helical peptide GPP10 acted as negative controls, and GFOGER as positive control. Mean A_450_± SEM is shown from *n* separate experiments, each performed in triplicate. (**A**) Wild-type α2 I domain (*n = *10). (**B**) α2 I E318W (*n = *12).

We previously used a solid-phase binding assay to investigate binding of α2 I E318W to the Collagen Toolkit III, which represents the entire triple helix of collagen III as a set of overlapping peptides [16]. In the present study, we have extended this analysis to the Collagen Toolkit II [20]. Wild-type α2 I domain bound a limited set of peptides from Toolkit II: II-7 and II-8 containing the GLOGER motif, and II-28 containing GFOGER (Fig. 1A). The E318W mutant bound the same three peptides with similar amplitude, and additionally bound (in order of decreasing amplitude) II-23, II-31, II-44, II-24, II-55, II-56, II-20, II-21 and II-18 (Fig. 1B). These emerging positive peptides include the motifs GMOGER, GQRGER and GAOGER, all reported as intermediate or weakly-binding ligands for integrin α2β1 [15].

**Figure 2:**
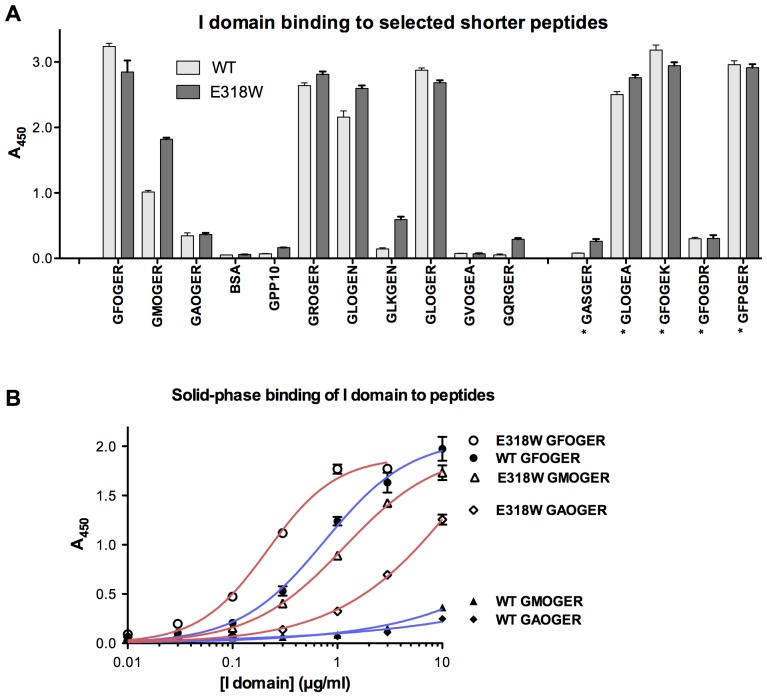
Binding of the integrin α2 I domain E318W mutant to selected peptides. (**A**) Wild-type and E318W α2 I domains were used in binding assays as described in the legend to Fig. 1, with shorter triple-helical peptides as substrates. The sequence of the peptides is indicated on the x-axis, where an asterisk indicates sequences not found in collagens II and III. Six paired experiments were performed, each in triplicate, and data represent mean A_450_± SEM. (**B**) Increasing concentrations of wild-type and E318W I domains were applied to GFOGER, GMOGER and GAOGER coatings, and binding was measured as above. Curves shown are the best fit non-linear single-site binding curves, obtained using GraphPad Prism 5 for Mac, of 3 replicates in a single experiment.

We also investigated the binding of α2 I E318W to a larger panel of triple-helical Gxx'GEx'' peptides than previously used [15], [16] (Fig. 2A). We observed a modest, but significant (p<0.001; 2-way ANOVA) increase in the binding of α2 I E318W across the board, compared with wild-type α2 I domain. In particular, binding of GMOGER stood out as doubling in amplitude (p<0.001; 1-way ANOVA), whilst amongst lower-affinity peptides, GLKGEN, GQRGER and GASGER showed several-fold increases in binding activity, albeit to low absolute amplitudes (p<0.001 for each; 1-way ANOVA). Both wild-type and E318W α2 I domain bound well to GFOGER, GROGER, GLOGEN and GLOGER, all present in collagens II and III, to GLOGEA and GFOGEK, occurring in other collagens, and to GFPGER, engineered into a bacterial collagen-like protein [21]. The relative increase in prominence of the lower-affinity Toolkit peptides in these solid-phase binding experiments may be interpreted as a relaxation in the ligand specificity of α2 I E318W, resembling that of integrin α2β1 in activated platelets [15], [16]. In binding experiments in which E318W and wild-type α2 I domains were applied in increasing dose to GFOGER, GMOGER and GAOGER, E318W showed a modest increase in affinity for GFOGER compared with wild-type and a substantially greater increase in affinity for GMOGER and GAOGER (Fig. 2B). The wild-type I domain bound these peptides to a greater extent than the negative control GPP-10 (not shown) only at the highest concentration tested.

**Binding of α2 I E318W to triple-helical peptides in solution.**

**Figure 3:**
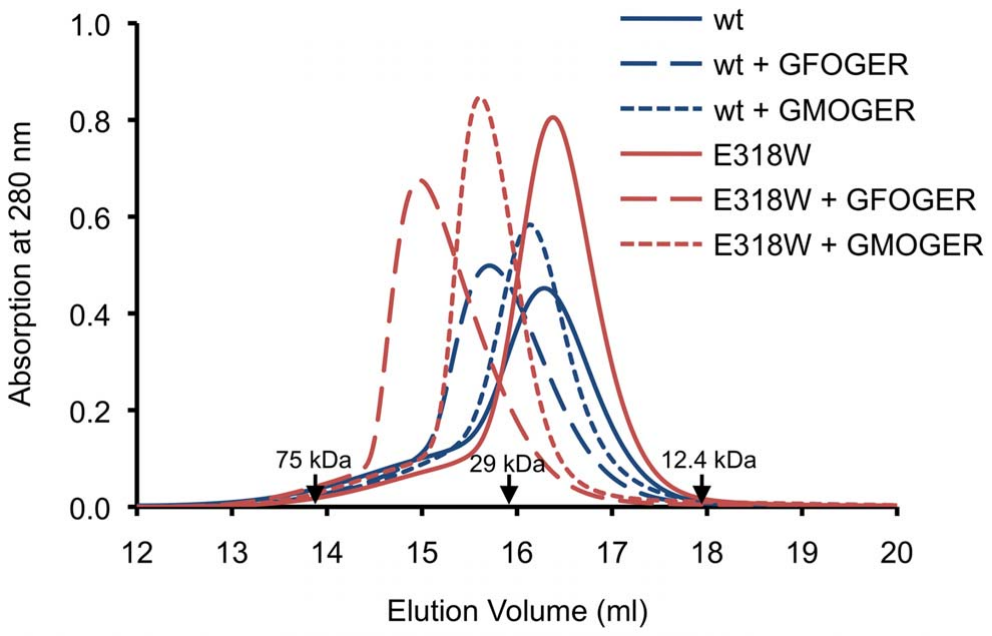
Analytical size exclusion chromatography of integrin α2 I domain-collagen complexes. The complexes were formed by incubating wild-type α2 I and α2 I E318W with the indicated peptides (peptide:I domain ≥2:1) and analysed on a Superdex 200 column. The peptides do not contribute to the absorbance at 280 nm. The elution volumes of three molecular mass standards are indicated by labelled arrows. The molecular mass of α2 I E318W is 25.1 kDa. The masses of the trimeric collagen peptides range from 5.7 to 5.9 kDa. The peaks at 14.9 and 15.6–15.7 ml are interpreted to contain, respectively, I domain-collagen complexes of 2:1 and 1:1 stoichiometry (see text).

As a prelude to crystallisation trials, we investigated the interactions of the wild-type and mutant α2 I domain with short collagen-like peptides ([GPO]_2_GxOGER[GPO]_3_,×  = F, M, A) in solution using analytical size exclusion chromatography (SEC). Because the collagen-like peptides do not absorb at 280 nm, they can be added in molar excess without obscuring the elution of the protein peak. The wild-type α2 I domain formed a complex with the GFOGER peptide, as evidenced by a 0.6 ml reduction in elution volume (Fig. 3). The apparent mass of the complex derived from the calibration of the SEC column is 31 kDa, in perfect agreement with the calculated mass of the previously crystallised 1:1 complex [9]. Addition of the GFOGER peptide to α2 I E318W caused a much greater reduction in elution volume (1.4 ml), suggestive of a larger complex containing two mutant I domains. The asymmetric peak profile suggests that some dissociation and re-association was occurring during the SEC run, which is further supported by the lower-than-expected apparent mass of the complex (44 kDa from calibration, 56.1 kDa calculated). The GMOGER peptide formed a stable 1:1 complex with α2 I E318W, but not with wild-type α2 I, and the GAOGER peptide did not interact with either I domain (not shown). We interpret the collective data as α2 I E318W having gained affinity for GMOGER and a second, less favourable, binding mode for GFOGER.

### Structure of the α2 I E318W-GFOGER Complex

**Figure 4:**
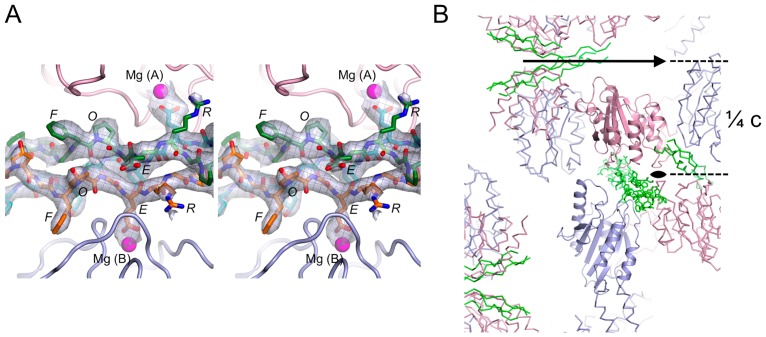
Electron density and crystal packing of the α2 I E318W-GFOGER complex. (**A**) Stereoview of the OMIT electron density of the collagen peptide. All collagen atoms were deleted from the final coordinate file and the partial structure was refined by simulated annealing to R_free_ = 0.37. The map shown is the resulting F_obs_-F_calc_ map contoured at 2 σ. The final collagen model is superimposed onto the map. The two α2 I E318W molecules are shown in pink (molecule A) and light blue (molecule B). Mg^2+^ ions are shown as magenta spheres. The collagen peptide is shown in orange (leading chain), green (middle chain) and cyan (trailing chain). The register of the three chains is unambiguously defined by the distinctive electron density of the GFO triplets and the clear absence of imino acids at the GER triplets. (**B**) Lattice interactions in the α2 I E318W-GFOGER crystal. The two crystallographically independent α2 I E318W molecules are shown in pink (molecule A) and light blue (molecule B), and the collagen peptide is shown in green. One asymmetric unit is shown in cartoon representation. The c-axis of the tetragonal crystals is vertical and the 2-fold axes along the ab-diagonals are indicated.

To reveal the details of the presumed 2:1 complex, we determined the structure of the α2 I E318W-GFOGER complex using crystals diffracting anisotropically to 3.04 Å resolution (Table 1). The unbiased electron density of the collagen peptide clearly indicated the register of all three chains (Fig. 4A) and the final residual difference density map also showed no evidence of register disorder (not shown). Model building and refinement was carried out with close reference to the high-resolution structure of the wild-type α2 I-GFOGER complex [9] and converged at a free R-factor of 0.298 for a model with excellent stereochemistry (Table 1).

**Table 1:** Crystallographic statistics.

Data collection	
Space group	P4_3_2_1_2
a, b, c (Å)	83.00, 83.00, 175.26
Resolution (Å)	29.4–3.04 (3.25–3.04)
R_merge_	0.111 (0.868)^a^
<I/σ(I)>	15.2 (2.9)
CC_1/2_ b	0.999 (0.929)
Completeness (%)	99.2 (96.5)
Multiplicity	13.4 (11.7)
Wilson B-factor (Å^2^)	79.3
**Refinement**	
Resolution (Å)	29.4–3.04
Reflections	12287
Protein atoms	3153
Solvent atoms or molecules	2 Mg^2+^, 1 Cl^–^, 1 bis-Tris
R_work_	0.246
R_free_	0.298
R.m.s. deviation bonds (Å)	0.003
R.m.s. deviation angles (°)	0.65
Average atomic B-factor (Å^2^)	83.9
Ramachandran plot (%)^c^	94.8, 4.9, 0.3

a Values in parentheses are for the highest resolution shell.

b Correlation coefficient between <I>of two independently processed half data sets [40].

c Residues in favoured, allowed and outlier regions of the Ramachandran plot [39].

**Figure 5:**
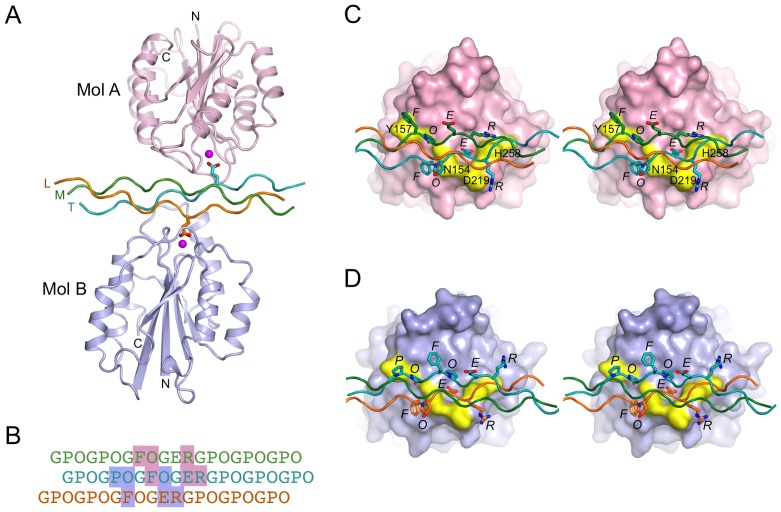
Crystal structure of the integrin α2 I E318W-GFOGER complex. (**A**) Cartoon representation of the asymmetric unit content. The two α2 I E318W molecules are shown in pink (molecule A) and light blue (molecule B). The collagen peptide is shown in orange (L, leading chain), green (M, middle chain) and cyan (T, trailing chain). Mg^2+^ ions are shown as magenta spheres. The two glutamic acid residues of the GFOGER peptide that are involved in I domain binding are shown as sticks. (**B**) Sequence of the GFOGER peptide and register of the L (orange), M (green) and T (cyan) chains. The footprint of the two α2 I E318W molecules is indicated by pink and light blue shading (molecules A and B, respectively). (**C**) Stereoview showing the interactions between α2 I E318W molecule A and the GFOGER peptide. The I domain is shown as a pink surface. The Mg^2+^ ion is in magenta, and selected residues important for collagen binding are in yellow and labelled. Selected collagen residues are shown as sticks and labelled. (**D**) Stereoview showing the interactions between α2 I E318W molecule B and the GFOGER peptide. The same colouring is used as in (C) except that the I domain is in light blue.

Consistent with the SEC data, the asymmetric unit of the crystals contains one GFOGER peptide interacting with two molecules of α2 I E318W, termed A and B (Fig. 5). The collagen-like peptide is straight and not bent as in the wild-type α2 I-GFOGER complex [9]. The glutamic acid residues of the trailing and leading collagen chains (defined as in [22]) interact, respectively, with the MIDAS Mg^2+^ ions of molecules A and B. The two α2 I E318W molecules in the complex are very similar to each other (r.m.s. deviation of 0.49 Å for 177 Cα atoms), as well as to the ligated conformation of the wild-type α2 I domain [9] (r.m.s. deviation of 0.58–0.59 Å). In molecule A, the C-terminal helix 7 has clear electron density for two helical turns, including the solvent-exposed side chain of W318. In molecule B, helix 7 has patchy electron density and W318 is disordered. The crystal lattice is formed from two types of 2-fold symmetric contacts, one involving the collagen peptide and α2 I domain residues 286–287 (highlighted in Fig. 4B) and the other involving only the α2 I domains at residues 228–233.

The interaction of α2 I E318W molecule A with the GFOGER peptide is essentially the same as described for wild-type α2 I [9]. The trailing collagen chain provides the crucial glutamic acid that coordinates the Mg^2+^ ion of the MIDAS; the same chain also provides the phenylalanine that makes van der Waals contacts with N154 and Q215, and the arginine that interacts electrostatically with D219 (Fig. 5B, C). The middle collagen chain provides the phenylalanine that makes van der Waals contacts with Y157 and L286, the hydroxyproline that donates a hydrogen bond to the peptide carbonyl oxygen of residue 154, and the arginine that stacks against H258 and is close to E256.

Molecule B also interacts with two of the three collagen chains, but because the three chains in a triple helix are not topologically equivalent, some of the interactions made by molecule A are not possible. The leading chain interacts with molecule B in the same way as the trailing chain with molecule A (Fig. 5B, D). In contrast, new interactions are formed by the second, trailing chain: a proline takes the place of the phenylalanine near Y157 and the arginine interacting with H258 is replaced by a hydroxyproline. These changes are expected to result in a weaker interaction, consistent with a smaller buried collagen surface area for molecule B (470 Å^2^) compared with molecule A (530 Å^2^).

We considered whether it is theoretically possible to achieve identical binding modes in a 2:1 complex. We modelled a complex in which I domain A is bound to the leading and middle chains, and I domain B is bound to the middle and trailing chains (the leading-middle and middle-trailing relationships are topologically equivalent). In contrast to the crystal structure, this modelled arrangement leads to clashes between the two I domains in a region that is key for allosteric regulation (residues 285–291) [9]. Thus, the crystal structure of the α2 I E318W-GFOGER complex represents the only sterically reasonable arrangement that can account for the observation of a 2:1 complex in solution (Fig. 3).

## Discussion

Our new binding data support the view that the E318W mutant represents an activated form of the α2 I domain that binds collagenous ligands in a largely conventional manner [15], [16], [18], [23]. In the solid-phase binding assay with Toolkit II, the E318W mutant displayed stronger discrimination of positive peptides than the wild-type α2 I domain and bound to several new peptides (Fig. 1). All the E318W-selective Toolkit peptides contain a GEx' triplet, consistent with the pivotal role of the glutamic acid in co-ordinating the Mg^2+^ ion of the MIDAS. Binding of α2 I E318W to shorter high-affinity peptides was similar to wild-type, probably because the assay was saturated. With lower affinity peptides, notably GMOGER, a substantial increase in binding of α2 I E318W was observed (Fig. 2). GFPGER, reported as able to bind integrin α2β1 but not α1β1 in recombinant human collagen [24], bound wild-type and E318W α2 I domain equally well despite the absence of the prolyl hydroxyl group. Notably, the α1β1-specific motif GVOGEA [12], either as a short peptide or within II-27, was not recognised by either form of α2 I domain.

In our recent co-crystallisation experiments with triple-helical peptides, we found analytical SEC to be a useful method to monitor protein-collagen peptide interactions in solution [22], [25], [26]. Using SEC, we detected a stable 1:1 complex of a short GMOGER peptide with α2 I E318W, but not with wild-type α2 I, again confirming that the E318W mutant is more active. Wild-type α2 I formed a 1:1 complex with a short GFOGER peptide, as expected [9]. Surprisingly, however, α2 I E318W appeared to form a 2:1 complex with the GFOGER peptide (Fig. 3).

The crystal structure of the α2 I E318W-GFOGER complex indeed revealed two α2 I domains, A and B, bound to the same triple helix (Fig. 5). A similar arrangement was recently described for the complex of the collagen chaperone Hsp47 with a triple-helical peptide [27]. I domain A of the α2 I E318W-complex binds to the trailing and middle chains of the GFOGER peptide, whilst the wild-type I domain bound to the middle and leading chains [9]. The integrin-collagen interactions are identical in both structures, however, because the trailing-middle and middle-leading combinations are topologically equivalent. We thus assign the interaction with I domain A as the high-affinity mode in the α2 I E318W-GFOGER complex. I domain B binds to the leading and trailing chains, and this interaction is assigned as the low-affinity mode. The collagen chain that contributes the MIDAS-binding glutamic acid makes identical interactions in the high- and low-affinity binding modes (Fig. 5). In contrast, the second (trailing) collagen chain in the low-affinity mode recapitulates only a single contact of the high-affinity binding mode (via the hydroxyproline of the preceding GPO triplet) and is lacking the interactions of the phenylalanine with Y157/L286 and the arginine with E256/H258. The peptide binding data suggest that the interaction with Y157/L286 contributes most to the binding affinity: the identity of residue×in the Gxx'GEx'' motif is highly correlated with affinity (F >L ≈ R >M >>A), whereas substitution of arginine in position x'' has only a small effect on binding (Fig. 2). Consistent with this interpretation, mutation of Y157 in integrin α2β1 has a stronger effect on collagen binding than mutation of either E256 or H258 [28]. Whether this 2:1 I domain–collagen interaction could be reproduced exactly in nature is open to question, since GFOGER occurs in the fibrillar collagens within different primary sequence settings than the flanking GPO triplets in our peptide. However, an adjacent GxO triplet, like that contributing to the trailing chain interaction revealed with I domain B, precedes several occurrences of GFOGER in other collagens, notably GFOGFOGER in α4(IV) and GPOGFOGER in α5(IV). The single-helix structure of collagen IV might permit simultaneous interaction with two I domains. Such speculation, although interesting, does not readily lend itself to experiment.

The low-affinity binding mode in the α2 I E318W-GFOGER complex suggests that a single GxOGER motif may support α2β1 binding, especially after activation of the cell in question, so long as the other collagen chain contacting the I domain does not cause steric hindrance. The markedly enhanced recognition of GMOGER-containing peptides by α2 I E318W suggests that this motif could also contribute to integrin-mediated cell adhesion. GMOGER occurs naturally in collagen α1(I), as a heterotrimer with GLOGER in the α2(I) chain, and in collagens α1(II), α1(III), α2(V) and α3(IX). In collagens V and IX, GMOGER is present without a corresponding motif in the other constituent α-chains. Recent advances in producing register-specific collagen heterotrimers [29], [30] may permit this hypothesis to be tested directly. Moreover, such a mode of binding might also operate in the fibrillar collagens where α-chains are sub-optimally exposed on the surface of a collagen fibre.

## Materials and Methods

### Expression Plasmids

The GST-tagged recombinant human α2 I domain-encoding plasmids (pGEX-2T-α2 I and pGEX-2T-E318W-α2 I) for binding studies were a generous gift from Dr D. Tuckwell (F2G Ltd, Manchester, UK). For crystallisation and SEC, the His-tagged E318W I domain ORF was cloned into the bacterial expression vector pDEST-N110 (a kind gift of Dr. M. Dyson) as described [31].

### Protein Production

The wild-type and E318W α2 I domains were produced in the same way for binding studies; recombinant proteins were expressed as described [12]. The procedure used to produce His-tagged E318W for crystallisation includes some modifications, and is detailed as follows. Origami™ *E. coli* cells (Novagen) were transformed with the respective expression plasmid and a 50 ml overnight culture used to inoculate 1 litre of Luria Broth medium containing 50 μg/ml ampicillin (Melford). After growing the culture for 2 h at 37°C, expression was induced with 0.5 mM isopropyl β-D-thiogalactopyranoside (Melford), and the culture grown overnight at 25°C. The cells were harvested by centrifugation for 20 min at 4500 *g* and the pellet was gently resuspended in 25 ml of ice-cold TES buffer (30 mM Tris-HCl pH 8.0, 1 mM EDTA, 20% sucrose) containing 1 tablet of EDTA-free protease inhibitor cocktail (Roche Applied Science), 50 mg of lysozyme (Sigma-Aldrich) and 25 mg of protamine sulphate (Sigma-Aldrich). The suspension was left on ice for 10 minutes and centrifuged for 10 min at 6000 *g* (4°C). The supernatant was collected and the pellet gently resuspended in 25 ml of ice-cold 5 mM MgSO_4_ containing 1 tablet of EDTA-free protease inhibitor cocktail, 50 mg of lysozyme and 25 mg of protamine sulphate. After 10 min on ice the suspension was centrifuged for 10 min at 6000*g* (4°C) and the pellet discarded. The pooled supernatants were centrifuged for 20 min at 16000*g* (4°C) and filtered through a 0.22 μm filter. Imidazole (Merck) was added to a final concentration of 50 mM and the solution loaded onto a 5 ml HisTrap FF column (GE Healthcare) equilibrated in PBS (phosphate buffered saline) using an ÄKTA Purifier system (GE Healthcare). The proteins were eluted with PBS containing 150 mM imidazole, concentrated using Vivaspin centrifugal devices (Sartorius) and further purified by SEC using a Superdex 75 10/300 GL column (GE Healthcare) and a running buffer consisting of 100 mM Tris-HCl pH 7.5, 150 mM NaCl and 2 mM MgCl_2_ (SEC buffer). The final protein yields were ∼10 mg/litre of culture.

### Peptide Synthesis

The general format of the peptides for solid-phase binding assay is GPC(GPP)_5_−[insert]-(GPP)_5_GPC. For Toolkit peptides, the insert contains 27 amino acids of primary collagen sequence, each subsequent peptide advancing 18 residues along the collagen triple-helical domain creating a 9−residue overlap between adjacent peptides in the set [16], [20]); shorter peptides contain a [Gxx'Gxx'] insert, with sequences as defined in Fig. 2, whilst the negative control, GPP10, lacks any insert. For crystallisation trials, peptides were made conforming to a shorter template, (GPO)_2_−[insert]-(GPO)_3_, the GPO flanking sequence providing greater thermal stability. Peptides, as C-terminal amides, were synthesised on TentaGel R-Ram resin using an Applied Biosystems Pioneer peptide synthesiser as described previously [16]. Shorter peptides were made using the same Fmoc chemistry in a CEM Liberty microwave synthesiser. In either case, fractions containing homogeneous product were identified by analytical HPLC on an ACEphenyl300 (5 mm) column, characterized by MALDI-TOF mass spectrometry, pooled and freeze-dried. Triple-helical conformation was confirmed by polarimetry.

### Toolkit and Shorter Peptide Binding Experiments

I domain adhesion was determined colorimetrically using a solid-phase binding assay, modified slightly from our previous studies [15], [16], [32] and specified below. Wells were coated using 1 μg peptide in 100 μl 0.01 M acetic acid for 1 h at 22°C on Immulon-2 HB 96-well plates (Thermo Life Sciences, Basingstoke, UK), and blocked for 1 h with 200 μl of TBS containing 50 mg/ml bovine serum albumin). Wells were washed four times with 200 μl of the adhesion buffer (TBS with 1 mg/ml bovine serum albumin) before adding 100 μl of adhesion buffer containing 10 μg/ml of recombinant GST I domains in the presence of either 2 mM MgCl_2_ or EDTA for 1 h at room temperature. Wells were washed five times with 200 μl of adhesion buffer containing MgCl_2_, before adding 100 μl of adhesion buffer containing Amersham anti-GST-HRP conjugate (RPN1236; GE Healthcare) at 1:10,000 dilution for 1 h at room temperature. After washing, colour was developed using an ImmunoPure TMB Substrate Kit (Pierce) according to the manufacturer’s instructions. Replacing Mg^2+^ with EDTA abolished binding (not shown). Data for short peptide binding were compared using 2-way ANOVA to establish the overall effect of the E318W mutation, and using repeated measures 1-way ANOVA for specific comparisons (Prism 5.0 for Mac, GraphPad).

### Analytical Size Exclusion Chromatography

The lyophilised triple-helical peptides (GFOGER, GMOGER, GAOGER) were dissolved in 500 μl of 1 mg/ml stock solutions of either wild-type or E318W α2 I domains. The amount of peptide was chosen so as to yield a greater than 2-fold molar excess of peptide. After incubation for 30 min at 4°C, the solutions were injected onto a Superdex 200 10/300 GL column and run at 4°C at a flow rate of 0.5 ml/min in SEC buffer.

### Crystallisation

The α2 I E318W-GFOGER complex was formed by dissolving 3.1 mg of lyophilised GFOGER peptide in 2 ml of a 3.5 mg/ml solution of α2 I E318W in SEC buffer (∼2:1 peptide:protein ratio). After incubation for 30 min at 4°C, the solution was subjected to SEC as in the analytical experiments. The fractions corresponding to the α2 I E318W-GFOGER complex were pooled, concentrated to 9 mg/ml and stored at 4°C overnight. A substantial precipitate was observed on the following day and pelleted by centrifugation. The clear supernatant was stable at 5.3 mg/ml and screened for crystallisation using a Mosquito nanolitre robot (TTP LabTech). Crystals grew after 2 days with 0.2 M MgCl_2_, 0.1 M bis-Tris pH 5.5, 25% PEG 3350 as precipitant. Crystals were flash-frozen in liquid nitrogen directly from the crystallisation drop.

### Data Collection and Structure Determination

Diffraction data were collected from a single α2 I E318W-GFOGER crystal at 100K on beamline I04 of the Diamond Light Source (Oxfordshire, UK) using a wavelength of 0.9795 Å and a CCD detector (ADSC). The diffraction data were integrated with XDS [33]. The diffraction limit was anisotropic, extending to<3.0 Å in the c* direction but limited to ∼3.5 Å in the a*b* plane. The integrated data were scaled and merged with AIMLESS, and converted to structure factor amplitudes with TRUNCATE [34]. The structure was solved by molecular replacement with PHASER [35] using as search model the wild-type α2 I-GFOGER complex [9]. The model was rebuilt with O [36], refined with CNS [37] and PHENIX [38], and validated with MOLPROBITY [39]. OMIT maps were used to verify the register of the three collagen chains (Fig. 4A) and the few changes with respect to the high-resolution structure of the α2 I-GFOGER complex [9]. The structure was refined with tight geometry restraints and restrained atomic B-factors. This treatment (which is physically more realistic than grouped B-factors) resulted in the lowest R_free_ and the smallest difference between R_work_ and R_free_. Mg^2+^-ligand distances were restrained to 2.07 Å. Difference density indicated the presence of a Mg^2+^-bound water molecule in each I domain; due to the low resolution of the diffraction data, water molecules were not included in the model, however. Crystallographic statistics are summarised in Table 1. The figures were made with PyMOL (www.pymol.org). Surface areas were calculated with AREAIMOL [34]. The coordinates of the α2 I E318W-GFOGER complex have been deposited in the Protein Data bank (entry code 4bj3).
